# Reference Ranges for Ascending Aorta Dimensions in Iranian Adults Assessed by 2D Echocardiography: Effect of Sex, Age, and Anthropometric Factors

**DOI:** 10.1155/crp/5904810

**Published:** 2025-10-28

**Authors:** Sadaf Agahi, Ehsan Goudarzi, Akram Sardari, Roya Sattarzadeh Badkoubeh, Mohammad Reza Eftekhari, Babak Geraiely, Farnoosh Larti

**Affiliations:** ^1^Department of Medicine, School of Medicine, Tehran University of Medical Sciences, Tehran, Iran; ^2^Department of Medicine, School of Medicine, Shahid Beheshti University of Medical Sciences, Tehran, Iran; ^3^Department of Cardiology, Imam Khomeini Hospital Complex, Tehran University of Medical Sciences, Tehran, Iran

**Keywords:** age, anthropometric, ascending aorta, dimensions, reference ranges, sex

## Abstract

**Background:**

Determining the normal ranges of the aortic dimension is essential in various populations. In this study, we aim to define the normal ranges for the sinus of Valsalva (SoV), the sinotubular junction (STJ), and the ascending aorta (AA) diameters using the Imam Khomeini Hospital Complex (IKHC) data registry of Iranian adults.

**Methods:**

This study was conducted on 2269 adult participants with left ventricular ejection fraction (LVEF) of more than 50%. Echocardiographic measurements were taken at the SoV, STJ, and AA levels. Subjects with any valvular stenosis and more than moderate insufficiency were excluded.

**Results:**

The normal range of SoV was found to be 24.5–38.9 mm in males and 21.7–34.9 mm in females. Additionally, the STJ diameters ranged from 19.7 to 32.5 mm and 18.0 to 29.6 mm in males and females, respectively. The AA measurements showed significant differences between sexes, ranging from 23.74 to 38.02 mm in males and 21.45 to 36.53 mm in females. Results also indicated that for every 10-year increase in age, the diameters of SoV, STJ, and AA increased by approximately 1.0, 0.8, and 1.6 mm, respectively.

**Conclusion:**

This study provided detailed echocardiographic reference values for aortic dimensions in the Iranian population and compared them across various age groups, genders, and body mass index (BMI) categories. Also, the findings emphasize the impact of aging on aortic values. The limited external validity of our single-center, hospital-based study suggests that future multicenter research is necessary to confirm our findings and improve their generalizability.

## 1. Introduction

Critical aortic diseases, such as aortic aneurysm and aortic dissection, lead to an estimated mortality rate of 2.5–3 deaths per 100,000 individuals and represent a major cause of cardiovascular morbidity and mortality. Although there have been remarkable improvements in cardiac diagnostic techniques over the last decades, the global burden of aortic diseases remains noticeable [1–3]. Aortic dimensions are fundamental in diagnosing and screening aortic diseases and are essential for effectively managing various aortic conditions [4, 5]. Abnormal aortic dimensions are associated with an elevated risk of dissection and serve as significant predictors of adverse cardiovascular events in patients with aortic disease [6–9]. Consequently, defining the normal range of aortic dimensions in various populations, in accordance with local guidelines, is essential for improving diagnostic accuracy, clinical decision-making, and predicting outcomes in patients with or at risk for aortic diseases.

Although advanced imaging modalities, such as computed tomography angiography (CTA) and magnetic resonance angiography (MRA), may offer greater sensitivity and specificity, transthoracic echocardiography (TTE) has been recommended as the first-line modality for assessing aortic measurements and disease evaluation (Class I Level B of recommendation). TTE is highly effective in evaluating patients with acute aortic syndromes (AAS), valve abnormalities, and overall cardiac function. Additionally, TTE plays an essential role in the longitudinal monitoring of aortic root and ascending aortic dilation, assuming these segments are properly observed [10–12]. Thus, TTE is a valuable tool for assessing aortic measurements and diagnosing abnormalities in both emergent and non-emergent clinical settings, particularly in the aortic root and proximal ascending aorta (AA) [10, 13–15].

This study aims to define the reference ranges for three important measures of the aorta: (1) sinus of Valsalva (SoV); (2) sinotubular junction (STJ); and (3) AA diameters in the Iranian adult population using TTE, to improve the precision of diagnosis and screening of aortic diseases.

## 2. Method

### 2.1. Study Participants

**Figure 1:**
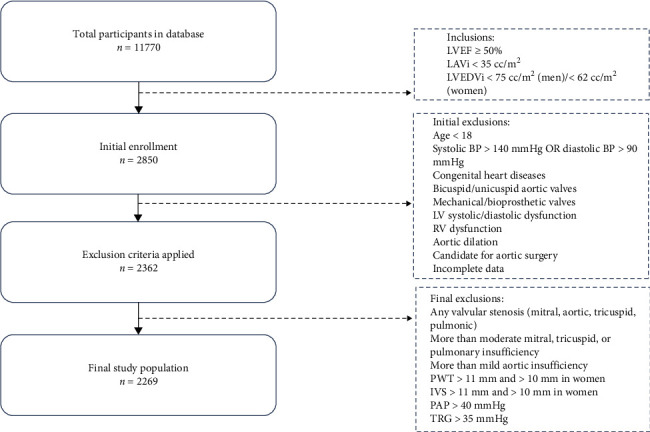
Flowchart of participant inclusion and exclusion.

This research utilized echocardiographic data from a large population-based database in our center. This web-based registry comprised 515 demographic, anthropometric, and echocardiographic records. The methodologies employed in this project have been previously explained [16]. From 11,770 participants in our database, 2850 adults with left ventricular ejection fraction (LVEF) of more than 50%, left atrium volume index (LAVi) of less than 35 cc/m^2,^ and left ventricular end-diastolic volume index (LVEDVi) of less than 75 cc/m^2^ in men and 62 cc/m^2^ in women were enrolled. Individuals under 18 and those with a systolic blood pressure (SBP) of more than 140 mmHg or a diastolic blood pressure (DBP) of more than 90 mmHg were excluded. Also, participants with any valvular stenosis (mitral stenosis, aortic stenosis, tricuspid stenosis, and pulmonic stenosis), more than moderate insufficiency of the mitral, tricuspid, and pulmonary valves, and more than mild aortic insufficiency were excluded. Posterior wall thickness (PWT) and interventricular septum (IVS) more than 11 mm in men and more than 10 mm in women, pulmonary artery pressure (PAP) more than 40 mm Hg, and tricuspid regurgitation gradient (TRG) more than 35 mmHg constituted the remaining exclusion criteria (Figure 1).

Furthermore, subjects with documented reports of any congenital heart disease, bicuspid or unicuspid aortic valves, mechanical or bioprosthetic valves, LV systolic or diastolic dysfunction, RV dysfunction, aortic dilation, candidates for any aortic surgery, and incomplete data were excluded. Finally, 2269 adult individuals who met the criteria were enrolled. To enhance our understanding of the subjects, we included demographic data such as gender, age, weight, height, body mass index (BMI), and body surface area (BSA) from the registry. Detailed echocardiographic measurements of aortic diameters, including SoV, STJ, and AA, were extracted. Due to the retrospective nature of the study, an informed consent waiver was granted by the ethics committee. This study adhered to the ethical guidelines of the 1975 Declaration of Helsinki and received approval from the Tehran University of Medical Sciences (IR.TUMS.IKHC.REC.1401.360).

### 2.2. Measurements

A team of four highly trained cardiologists with a fellowship in echocardiography conducted all echocardiographic examinations. All echocardiographic measurements were performed by four dedicated, expert echocardiographers who strictly adhered to a standardized, predefined institutional protocol. This rigorous, unified approach, combined with the high level of expertise of the observers, ensured the consistency and quality of all data collected. The echocardiographic machines in our laboratory were Philips Affinity 50, Philips EPIQ7, and Philips CVx. We also followed the established guidelines and recommendations of ASE/ECAVI [17] for echocardiographic measurements. The AA was measured at the end-diastolic frame from the leading edge of the anterior wall to the leading edge of the posterior wall, identifying the largest diameter. The SoV was the widest part of the sinus measured using the leading edge-to-leading edge method. The STJ was also measured using the leading edge-to-leading edge method at end-diastole at the level of transition of SoV into the tubular part of the AA. The BSA was calculated for all participants using the formula (height + weight - 60)/100. LVEF was measured by eyeball assessment. Tricuspid regurgitation velocity (TRV) was calculated by taking the square root of the TRG divided by 2. The AA was indexed by dividing its measurement by the BSA. Obesity was defined as BMI ≥ 35 kg/m^2^.

### 2.3. Statistical Analysis

The participants' baseline characteristics and echocardiographic data are presented as mean ± standard deviation (SD) for continuous variables and frequency (percentage) for categorical variables. Steam-and-leaf and normality Q–Q plots tested the normal distribution of parameters. All the variables in this study had normal distribution, so mean ± 2SD was calculated to express the reference (normal) ranges. Independent sample *t*-tests were used to compare the means of categorical groups. Comparisons of means across more than two groups were performed using analysis of variance (ANOVA). Correlation analysis and linear regression were employed to investigate the association between aortic dimensions and demographic parameters. We utilized Stata Version 17.0 for all the analyses, with a significance level of 0.05. We also used Adobe Illustrator Version 27.1.1 to create and edit the figures.

## 3. Results

A total of 2269 subjects were recruited for this study, with 39.3% male gender. The mean age of the participants was 45 years (SD = 14). Anthropometric measures, including BMI, BSA, height, and weight, were significantly higher in males compared to females. However, SBP and DBP did not differ significantly between sexes, with respective means of 118 and 74 mmHg across all subjects (Table 1).

Table 2 demonstrates all means and ranges of echocardiographic measures. The means of SoV, STJ, and AA were significantly higher in males, with respective means of 31.7, 26.1, and 30.88 mm, compared to 28.3, 23.8, and 28.99 mm in females. In males, LV-PW, LV-IVS, LVEDVi, TRG, and TRV were significantly higher. Conversely, peak and mean gradients were significantly higher in the female group.

**Figure 2:**
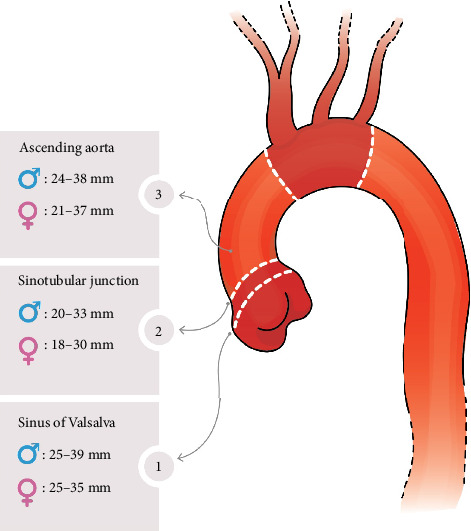
Reference (normal) ranges of sinus of Valsalva (SoV), sinotubular junction (STJ), and ascending aorta (AA) diameters in the Iranian population1.

The reference (normal) ranges of the SoV, STJ, and AA were meticulously calculated using mean ± 2SD and are presented in Table 3. For males, the normal range of SoV was found to be 24.5–38.9 mm, while for females, it was slightly narrower at 21.7–34.9 mm. Similarly, the STJ measurements ranged from 19.7 to 32.5 mm in males and 18.0–29.6 mm in females. The AA dimensions also showed a distinct difference between sexes, with a range of 23.74–38.02 mm in males and 21.45–36.53 mm in females. In Table 3, the normal ranges of indexed aorta dimensions are also provided.  Figure 2 summarizes normative ranges of SoV, STJ, and AA in males and females.

Our analysis compared the STJ, SoV, and AA dimensions between obese and normal-weight subjects (Table 4). In males, these measures did not show significant differences. However, in females, SoV, STJ, and AA were significantly larger in obese individuals.

Additionally, we examined these dimensions across these age groups: 18–40, 41–65, and over 65 years (Table 5). SoV, STJ, and AA dimensions increased significantly with age in both males and females, highlighting the impact of aging on aortic dimensions.

We also compared the dimensions of the SoV, STJ, and AA across different SBP groups (Table 6). The results revealed that SoV exhibited a rising trend with higher SBP, although these differences were not statistically significant. The STJ measurements did not follow a consistent pattern across SBP groups. In contrast, the AA dimensions significantly increased with rising SBP, indicating a clear correlation between higher SBP and larger AA measurements.

In the correlation analysis, age was significantly associated with the SoV, STJ, and AA diameters, with respective Pearson correlations of 0.36, 0.35, and 0.55. In the linear regression analysis of age with AA, the association was significant, with a coefficient of 0.157 and an R-squared value of 0.305. Similarly, the STJ demonstrated a significant association with age, with a coefficient of 0.082 and an R-squared value of 0.122. Lastly, the association of age with the SoV also reached statistical significance, with a coefficient of 0.099 and an R-squared value of 0.127.

In linear regression analysis of BSA with SoV, STJ, and AA, the association was significant (*p* value < 0.001) with respective coefficients of 6.77, 4.83, and 4.87. The R-squared values were 0.143, 0.101, and 0.073, respectively.

## 4. Discussion

Data collected from 2269 adults provides a high-power analysis to evaluate the normal ranges of the root and AA. The hallmark of the presenting study is the uniformity of data acquisition with experienced operators. These are essential landmarks for better insights into aortic diseases in the Iranian adult population. To the best of our knowledge, this is the first study to report normal values of SoV and STJ diameters in the Middle East, and it represents one of the most extensive studies globally in terms of the number of participants.

### 4.1. Mean Aortic Dimensions: A Comparative Perspective

**Figure 3:**
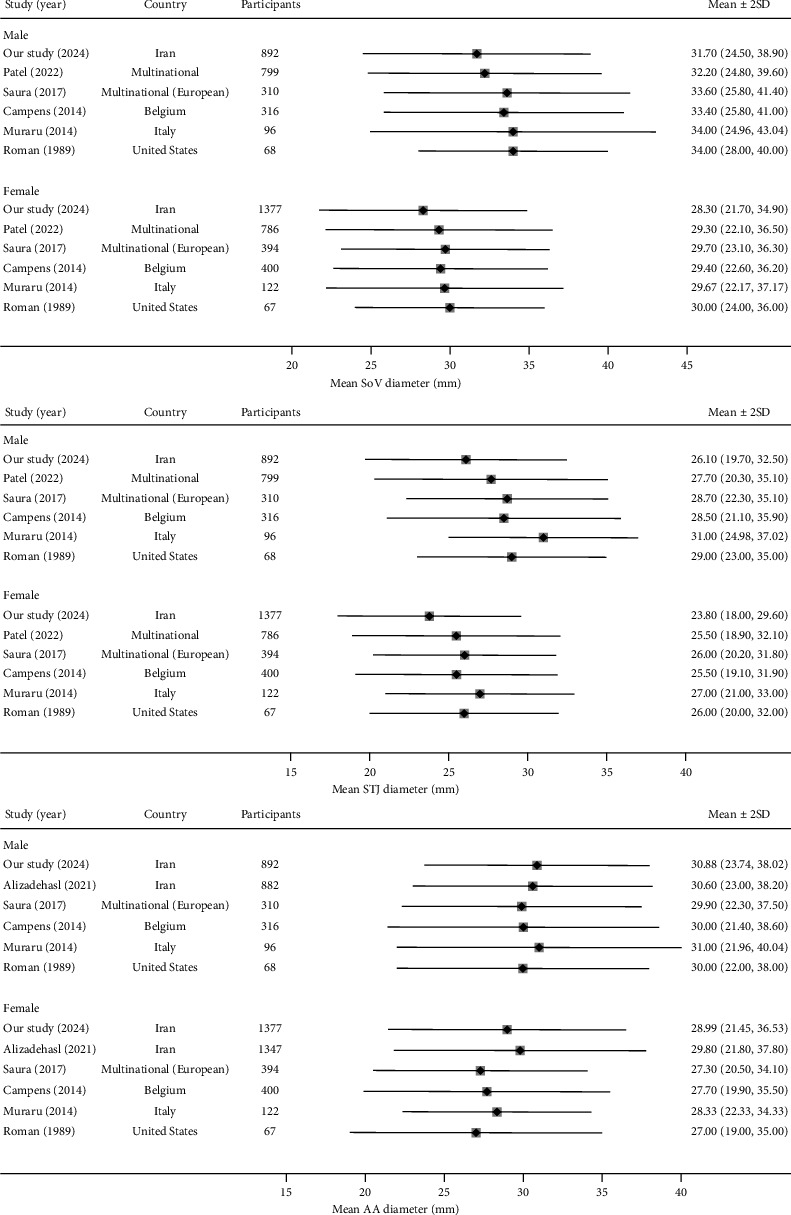
Cross-study comparisons of the sinus of Valsalva (SoV), the sinotubular junction (STJ), and ascending aorta (AA) diameter measurements.

Based on our study, the mean SoV diameter in the assessed population is 31.7 mm for males and 28.3 mm for females, with an overall mean diameter of approximately 29.6 mm. In a multicenter study conducted across 15 countries in 2022, Patel et al. [18] evaluated SoV and STJ diameters using 2D-TTE to determine aortic normal ranges and compared results between males and females across races. Compared to our study, the results of Patel et al. showed a higher mean SoV diameter in both males and females by 0.5 mm and 1 mm, respectively (Figure 3). This pattern is also observed when comparing our results to other studies; the results of [19, 19], conducted in multiple European countries, showed an increase of 1.9 mm in males and 1.4 mm in females in SoV diameter compared to our mean values. The greatest difference compared to our results was observed in Roman et al. [13] in the United States, which reported increases of 2.3 mm in males and 1.7 mm in females in the SoV diameter. However, Patel et al. [18] demonstrated that differences in aortic dimensions were observed by race, with the mean SoV diameter in Asians being significantly lower than in White and Black populations. The study reported a mean SoV diameter of 32.2 mm in Asian men and 29.0 mm in Asian women, with an overall mean of 29.6 mm, which is highly consistent with our results.

Additionally, LaBounty et al. 2019 [20] showed that the Asian population had a lower SoV diameter compared to the White population but was similar to Black and Hispanic populations. Regarding the STJ landmark, our research showed mean diameters of 26.1 mm and 23.8 mm in males and females, respectively, with an average of 24.6 mm. The findings also indicated a lower mean diameter than other studies [13, 18, 19, 21, 22]. The investigations by Patel et al. [18] also showed that the Asian population had a significantly lower STJ diameter compared to Black and White populations. These findings emphasize that the normal ranges for aortic measurements should be defined based on race and ethnicity.

Alizadehasl et al. [23] conducted a study in 2021 to evaluate normal values for echocardiographic findings in Iran. Their results highlighted a mean AA diameter of 30.6 mm in males and 29.8 mm in females, which aligns closely with our findings of 30.88 mm and 28.99 mm in males and females, respectively. However, the results are accurate, as the study does not exclude subjects with valvular stenosis, regurgitation, or hypertension. Also, patients with LV systolic/diastolic dysfunctions have been included, preventing the results from showing normal values accurately. Our findings are in strong agreement with some previous studies on AA diameters in males; however, they showed a slight difference in females, the significance of which should be evaluated in future research [13, 19, 21, 22].

### 4.2. Determining Upper Normal Ranges for Aortic Dimensions

An aneurysm is defined as an increase in aortic diameter exceeding 1.5 times the expected size. In contrast, aortic dilation refers to an aortic diameter greater than the 95th percentile for a normal individual of the same age, sex, and body size [10, 24]. In 2019, the age-standardized death rate due to aneurysms was reported as 2.21 per 100,000 persons [25]. Therefore, defining the upper normal ranges of aortic measurements in each population is essential for diagnosing and monitoring aortic dilation and aneurysms. The “2024 ESC Guidelines for the management of peripheral arterial and aortic diseases” defined upper normal values for SoV as 40 mm for males and 34 mm for females and for STJ as 38 mm for males and 33 mm for females.

Regarding AA diameter, the upper normal range was defined as 40 mm for males and 36 mm for females [11]. In the Iranian population, our data demonstrated upper normal ranges of 39 mm and 35 mm for SoV diameters and 38 and 37 mm for AA diameters in males and females, respectively, which are almost compatible with the ESC guidelines. However, the upper normal ranges for STJ diameters were lower compared to the ESC guidelines, at 33 mm for males and 30 mm for females (Figure 3). These alterations suggest that careful consideration should be given to diagnosing aneurysms in the STJ, and further studies are needed to propose precise cutoff points for detecting aortic diseases across different populations.

### 4.3. Impact of Demographic Variables on Aortic Measurements

The association between BSA and root and AA diameters has been assessed in several studies among different populations [18, 26, 27]. According to the European Association of Echocardiography (EAE) recommendations for echocardiography in aortic diseases, the relationship between aorta size and BSA should be considered when defining normal ranges [15]. The latest ESC 2024 guidelines highlight BSA as the primary method for adjusting aortic dimensions according to an individual's body size. In cases of extreme body weight, whether very low or very high, the use of height for adjusting aortic measurements is recommended. Additionally, an increased risk of aortic aneurysm enlargement (AAE) has been noted with increasing diameters indexed to BSA and height (13). The assessed normal ranges of indexed aortic dimensions based on our data are shown in Table 3. Some studies have shown that body weight influences aortic dimensions [28]. Therefore, certain researchers have proposed indexing aortic measurements by height to minimize the impact of overweight on BSA [29]. Our study demonstrates a significant relationship between BSA and all three SoV, STJ, and AA diameters, with each 0.1 m^2^ increase in BSA corresponding to an approximate 0.5 to 0.7 mm increase in aortic diameters at the mentioned landmarks.

We also compared the SoV, STJ, and AA diameters between individuals with a BMI below and above 35 kg/m^2^. The results revealed significant differences in SoV, STJ, and AA diameters between the two BMI groups in women. However, none of the SoV, STJ, or AA diameters showed significant differences between the BMI groups in men.

Age is another demographic parameter associated with aortic dimensions, with research showing that aortic size increases throughout life [6]. Our findings confirmed this evidence, revealing that with every 10-year increase in age, the SoV, STJ, and AA diameters enlarged by approximately 1.0, 0.8, and 1.6 mm, respectively.

Using population-specific ranges allows for earlier and more accurate identification of aortic dilation. Standard thresholds may miss early-stage aneurysms in populations with naturally smaller aortic dimensions. By using a more tailored reference range, clinicians can detect abnormal enlargement sooner, enabling timely surveillance and intervention. This proactive approach can prevent the progression of the aneurysm to a size that poses a higher risk of rupture or dissection. A patient with an aortic dimension at the upper limit of the population-specific normal range (but below the universal guideline) could be placed on a more frequent imaging surveillance schedule and receive more aggressive medical therapy to control blood pressure (BP), thereby mitigating the risk of further dilation. Conversely, a universal threshold might lead to unnecessary anxiety or interventions in populations with naturally larger aortas. For instance, if the average aortic size is slightly larger, a measurement that is near the standard threshold might not be as clinically significant. Using a tailored range helps clinicians avoid overtreating patients who fall within their population's normal spectrum, thus conserving healthcare resources and preventing unneeded surgical risks.

## 5. Limitations

Although some research has shown that rising BP and hypertension are associated with aortic root enlargement and dissection, BP as a factor in aortic root enlargement continues to be controversial [30]. This study investigated the relationship between SBP and SoV, STJ, and AA diameters in patients with SBP below 140 mmHg. While the results for STJ and AA diameters were significant, no increasing trend was observed between rising SBP and the diameters of SoV, STJ, or the AA. The main limitation of our study was the inaccessibility to the patient's cardiovascular risk factors and family history. The data obtained for each patient were based only on the evaluations done before echocardiography, which prevented us from excluding individuals with hypertension or other potentially influential comorbidities. Another limitation of our study is that our echocardiographically normal data were not directly gathered from the community but from participants attending a tertiary care center. Its single-center, hospital-based design limits the external validity of our findings, as patients treated at this specialized institution may represent a more severe or complex disease spectrum than those seen in community settings. Future multicenter studies are therefore needed to confirm our results and improve the generalizability of our findings across a more diverse patient population.

## 6. Conclusion

**Figure 4:**
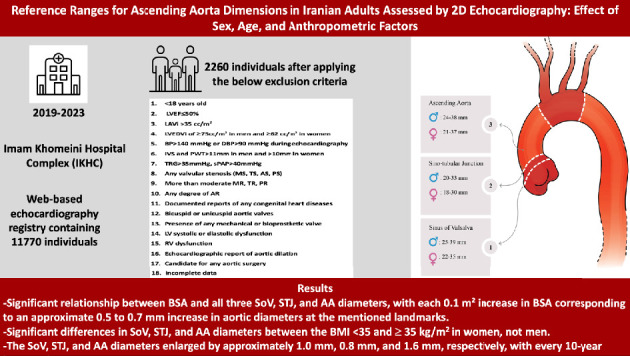
Summary of the study design and results.

In this study, we assessed the diameters of the SoV, STJ, and AA in 2269 patients and proposed normal ranges for aortic measurements. Additionally, we evaluated the impact of age, BSA, BMI, and BP on these aortic dimensions within the studied population. The results provide valuable insights that could enhance the detection and monitoring of diseases affecting the aortic root and AA. By integrating these findings into local guidelines, healthcare professionals can better predict, diagnose, and treat critical aortic diseases, ultimately reducing cardiovascular mortality. A summary of the study design and results is provided in Figure 4.

## Figures and Tables

**Table 1:** Baseline demographic characteristics.

Demographics	All	Male	Female
*N*	2269	892 (39.3%)	1377 (60.7%)
Age (years)	45 ± 14 (18–89)	46 ± 14 (18–89)	44 ± 13^∗^ (18–85)
Height (cm)	167 ± 9 (136–210)	174 ± 8 (136–210)	162 ± 7^∗^ (137–193)
Weight (kg)	74 ± 15 (34–150)	78 ± 15 (38–143)	72 ± 15^∗^ (34–150)
BMI (kg/m^2^)	26.67 ± 5.21 (12.91–60.85)	25.65 ± 4.42 (13.44–44.08)	27.30 ± 5.54^∗^ (12.91–60.85)
BSA (m^2^)	1.81 ± 0.21 (1.21–2.65)	1.92 ± 0.20 (1.31–2.65)	1.73 ± 0.18^∗^ (1.21–2.58)
SBP (mmHg)	118 ± 12 (80–140)	118 ± 12 (84–140)	117 ± 12 (80–140)
DBP (mmHg)	74 ± 9 (40–90)	75 ± 9 (43–90)	74 ± 10 (40–90)

Abbreviations: BMI, body mass index; BSA, body surface area; DBP, diastolic blood pressure; SBP, systolic blood pressure.

^∗^Values in parentheses show the lower and upper range of the parameter.

^∗^ *p* value < 0.05 men versus women.

**Table 2:** Baseline echocardiographic measurements.

	All	Male	Female	*p* value
LVEF (%)	55 ± 3 (50–65)	54 ± 3 (50–65)	55 ± 2^∗^ (50–65)	< 0.001
Sinus of Valsalva (mm)	29.6 ± 3.8 (19.0–45.0)	31.7 ± 3.6 (20.0–45.0)	28.3 ± 3.3^∗^ (19.0–42.0)	< 0.001
Sinus of Valsalva/height (mm/cm)	0.18 ± 0.02 (0.12–0.26)	0.18 ± 0.02 (0.12–0.26)	0.18 ± 0.02^∗^ (0.12–0.25)	< 0.001
Sinus of Valsalva/BMI (mm ∗ m^2^/kg)	1.15 ± 0.25 (0.44–2.42)	1.27 ± 0.24 (0.63–2.42)	1.07 ± 0.23^∗^ (0.44–2.25)	< 0.001
Sinus of Valsalva/BSA (mm/m^2^)	16.54 ± 2.26 (9.86–26.49)	16.65 ± 2.28 (10.19–26.16)	16.47 ± 2.23 (9.86–26.49)	0.66
Sinotubular junction (mm)	24.6 ± 3.2 (12.0–35.0)	26.1 ± 3.2 (17.0–35.0)	23.8 ± 2.9^∗^ (12.0–35.0)	< 0.001
Sinotubular junction/height (mm/cm)	0.15 ± 0.02 (0.07–0.23)	0.15 ± 0.02 (0.09–0.21)	0.15 ± 0.02^∗^ (0.07–0.23)	< 0.001
Sinotubular junction/BMI (mm ∗ m^2^/kg)	0.96 ± 0.21 (0.39–1.88)	1.04 ± 0.19 (0.56–1.88)	0.90 ± 0.19^∗^ (0.39–1.86)	< 0.001
Sinotubular junction/BSA (mm/m^2^)	13.76 ± 1.98 (6.67–22.73)	13.70 ± 1.99 (9.01–20.76)	13.81 ± 1.97 (6.67–22.73)	0.214
Ascending aorta (mm)	29.71 ± 3.81 (17.00–40.00)	30.88 ± 3.57 (21.00–40.00)	28.99 ± 3.77^∗^ (17.00–40.00)	< 0.001
Ascending aorta/height (mm/cm)	0.18 ± 0.02 (0.11–0.27)	0.18 ± 0.02 (0.11–0.24)	0.18 ± 0.02 (0.11–0.27)	0.119
Ascending aorta/BMI (mm ∗ m^2^/kg)	1.15 ± 0.23 (0.44–2.29)	1.23 ± 0.22 (0.63–2.29)	1.09 ± 0.22^∗^ (0.44–2.26)	< 0.001
Ascending aorta/BSA (mm/m^2^)	16.60 ± 2.46 (10.10–28.17)	16.23 ± 2.38 (10.19–24.63)	16.84 ± 2.48^∗^ (10.10–28.17)	< 0.001
LV-PW (mm)	8.4 ± 1.5 (0.6–11.0)	8.7 ± 1.5 (0.7–11.0)	8.2 ± 1.4^∗^ (0.6–10.0)	< 0.001
LV-IVS (mm)	8.7 ± 1.5 (0.7–11.0)	9.1 ± 1.5 (0.8–11.0)	8.5 ± 1.4^∗^ (0.7–10.0)	< 0.001
LVEDVi (mL/m^2^)	47.6 ± 9.4 (18.5–74.0)	50.4 ± 10.3 (18.5–74.0)	45.8 ± 8.3^∗^ (18.9–61.5)	< 0.001
Aortic peak gradient (mmHg)	7.55 ± 4.33 (2.00–48.00)	7.13 ± 4.74 (2.00–48.00)	7.81 ± 4.04^∗^ (2.00–47.00)	< 0.001
Aortic mean gradient (mmHg)	4.25 ± 3.00 (1.00–35.00)	4.02 ± 3.25 (1.00–35.00)	4.40 ± 2.84^∗^ (1.50–33.00)	0.006
TRG (mmHg)	22.7 ± 4.6 (9.0–35.0)	23.1 ± 4.6 (11.0–35.0)	22.6 ± 4.7^∗^ (9.0–35.0)	0.31
TRV (m/s)	2.37 ± 0.25 (1.50–2.96)	2.39 ± 0.24 (1.66–2.96)	2.36 ± 0.25^∗^ (1.50–2.96)	0.26

Abbreviation: LVEF, left ventricular ejection fraction.

^∗^Values in parentheses show the lower and upper range of the parameter.

^∗^ *p* value < 0.05 men versus women.

**Table 3:** Projected normative ranges of SoV, STJ, and AA and indexed values according to height, BSA, and BMI.

	Male	Female
Sinus of Valsalva (mm)	24.5–38.9	21.7–34.9
Sinus of Valsalva/height (mm/cm)	0.14–0.22	0.14–0.22
Sinus of Valsalva/BMI (mm ∗ m^2^/kg)	0.79–1.75	0.61–1.53
Sinus of Valsalva/BSA (mm/m^2^)	12.09–21.21	12.01–20.93
Sinotubular junction (mm)	19.7–32.5	18.0–29.6
Sinotubular junction/height (mm/cm)	0.11–0.19	0.11–0.19
Sinotubular junction/BMI (mm ∗ m^2^/kg)	0.66–1.42	0.52–1.28
Sinotubular junction/BSA (mm/m^2^)	9.72–17.68	9.87–17.75
Ascending aorta (mm)	23.74–38.02	21.45–36.53
Ascending aorta/Height (mm/cm)	0.14–0.22	0.14–0.22
Ascending aorta/BMI (mm ∗ m^2^/kg)	0.79–1.67	0.65–1.53
Ascending aorta/BSA (mm/m^2^)	11.47–20.99	11.88–21.80

**Table 4:** Comparison of measurements of SoV, STJ, and AA across age groups.

	Male				Female			
	18–40 y	41–65 y	> 65 y	*p* value	18–40 y	41–65 y	> 65 y	*p* value
*N* (%)	328 (37.3%)	481 (54.7%)	70 (8.0%)	NA	583 (42.9%)	712 (52.4%)	65 (4.8%)	NA
Sinus of Valsalva (mm)	30.2 ± 3.4	32.6 ± 3.3	33.4 ± 3.9	< 0.001	27.1 ± 3.0	29.2 ± 3.1	30.1 ± 3.7	< 0.001
Sinotubular junction (mm)	24.6 ± 3.0	26.9 ± 2.9	27.3 ± 3.2	< 0.001	22.7 ± 2.9	24.4 ± 2.6	25.6 ± 2.9	< 0.001
Ascending aorta (mm)	28.31 ± 4.30	32.24 ± 3.80	33.37 ± 5.21	< 0.001	26.93 ± 3.15	30.38 ± 3.85	33.29 ± 3.42	< 0.001

**Table 5:** Comparison of measurements of SoV, STJ, and AA in normal and obese subjects.

	Male			Female		
	BMI < 35 kg/m^2^	BMI ≥ 35 kg/m^2^	*p* value	BMI < 35 kg/m^2^	BMI ≥ 35 kg/m^2^	*p* value
*N* (%)	846 (95.8%)	25 (3.1%)	NA	1240 (91.9%)	116 (8.1%)	NA
Sinus of Valsalva (mm)	31.7 ± 3.6	31.9 ± 4.0	0.830	28.3 ± 3.3	29.0 ± 3.0	0.010
Sinus of Valsalva/height (mm/cm)	0.18 ± 0.02	0.18 ± 0.02	0.832	0.17 ± 0.02	0.18 ± 0.02	< 0.001
Sinus of Valsalva/BMI (mm ∗ m^2^/kg)	1.28 ± 0.23	0.85 ± 0.14	< 0.001	1.11 ± 0.21	0.75 ± 0.10	< 0.001
Sinus of Valsalva/BSA (mm/m^2^)	16.73 ± 2.22	14.11 ± 2.64	< 0.001	16.65 ± 2.17	14.58 ± 2.04	< 0.001
Sinotubular junction (mm)	26.1 ± 3.2	26.0 ± 2.6	0.968	23.7 ± 2.9	24.4 ± 2.6	0.005
Sinotubular junction/height (mm/cm)	0.15 ± 0.02	0.15 ± 0.02	0.974	0.15 ± 0.02	0.15 ± 0.02	< 0.001
Sinotubular junction/BMI (mm ∗ m^2^/kg)	1.05 ± 0.19	0.70 ± 0.10	< 0.001	0.93 ± 0.18	0.63 ± 0.08	< 0.001
Sinotubular junction/BSA (mm/m^2^)	13.77 ± 1.95	11.54 ± 2.19	< 0.001	13.96 ± 1.93	12.25 ± 1.71	< 0.001
Ascending aorta (mm)	30.89 ± 3.59	30.60 ± 3.043	0.702	28.88 ± 3.74	30.19 ± 3.71	< 0.001
Ascending aorta/height (mm/cm)	0.18 ± 0.02	0.18 ± 0.02	0.590	0.18 ± 0.02	0.19 ± 0.02	< 0.001
Ascending aorta/BMI (mm ∗ m^2^/kg)	1.24 ± 0.21	0.82 ± 0.12	< 0.001	1.12 ± 0.20	0.78 ± 0.12	< 0.001
Ascending aorta/BSA (mm/m^2^)	16.31 ± 2.34	13.47 ± 2.53	< 0.001	17.00 ± 2.41	15.19 ± 2.54	< 0.001

**Table 6:** Comparison of measurements of SoV, STJ, and AA across systolic blood pressure groups.

SBP (mmHg)	≤ 100	101–110	111–120	121–130	131–140	*p* value
*N* (%)	259 (12.9%)	448 (22.3%)	578 (28.8%)	466 (23.2%)	255 (12.7%)	NA
Sinus of aorta (mm)	29.4 ± 3.9	29.6 ± 3.7	29.5 ± 3.8	29.9 ± 3.7	29.6 ± 3.6	0.277
Sinotubular junction (mm)	24.6 ± 3.5	24.4 ± 3.3	24.5 ± 3.1	25.0 ± 3.1	24.4 ± 3.0	0.044
Ascending aorta (mm)	29.1 ± 3.7	29.4 ± 3.7	29.5 ± 3.8	30.3 ± 3.8	30.0 ± 3.8	< 0.001

## Data Availability

Data supporting the study results are available and can be requested upon reasonable request from the corresponding author.

## Nomenclature

AA: Ascending aorta
AAS: Acute aortic syndromes
BMI: Body mass index
BSA: Body surface area
CTA: Computed tomography angiography
DBP: Diastolic blood pressure
EAE: European Association of Echocardiography
IKHC: Imam Khomeini Hospital Complex
LAVi: Left atrium volume index
LVEF: Left ventricular ejection fraction
LVEDVi: Left ventricular end-diastolic volume index
MRA: Magnetic resonance angiography
PAP: Pulmonary artery pressure
PWT: Posterior wall thickness
SBP: Systolic blood pressure
SD: Standard deviation
SoV: Sinus of Valsalva
STJ: Sinotubular junction
TRG: Tricuspid regurgitation gradient
TRV: Tricuspid regurgitation velocity
TTE: Transthoracic echocardiography

## Endnotes

^1^The ranges have been rounded up for better illustration.
